# Interaction between the expression of hsa_circRPRD1A and hsa_circHERPUD2 and classical coronary risk factors promotes the development of coronary artery disease

**DOI:** 10.1186/s12920-023-01540-9

**Published:** 2023-06-14

**Authors:** Shu He, Yahong Fu, Chengcheng Li, Xiongkang Gan, Yanjun Wang, Hanxiao Zhou, Rongli Jiang, Qian Zhang, Qiaowei Jia, Xiumei Chen, En-Zhi Jia

**Affiliations:** 1 Department of Cardiovascular Medicine, the First Affiliated Hospital of Nanjing Medical University, Guangzhou Road 300, Nanjing, Jiangsu Province 210029 China; 2 Department of Geriatric, the First Affiliated Hospital of Nanjing Medical University, Guangzhou Road 300, Nanjing, Jiangsu Province 210029 China; 3 Department of Cardiovascular Medicine, Liyang People’s Hospital, Liyang, Jiangsu province 213300 China

**Keywords:** Coronary artery disease, Circular RNA, RNA sequencing, Crossover analysis, Peripheral blood mononuclear cells

## Abstract

**Background:**

Recent studies suggest that classical coronary risk factors play a significant role in the pathogenesis of coronary artery disease. Our study aims to explore the interaction of circRNA with classical coronary risk factors in coronary atherosclerotic disease.

**Method:**

Combined analysis of RNA sequencing results from coronary segments and peripheral blood mononuclear cells of patients with coronary atherosclerotic disease was employed to identify critical circRNAs. Competing endogenous RNA networks were constructed by miRanda-3.3a and TargetScan7.0. The relative expression quantity of circRNA in peripheral blood mononuclear cells was determined by qRT-PCR in a large cohort including 256 patients and 49 controls. Spearman’s correlation test, receiver operating characteristic curve analysis, multivariable logistic regression analysis, one-way analysis of variance, and crossover analysis were performed.

**Results:**

A total of 34 circRNAs were entered into our study, hsa_circRPRD1A, hsa_circHERPUD2, hsa_circLMBR1, and hsa_circDHTKD1 were selected for further investigation. A circRNA-miRNA-mRNA network is composed of 20 microRNAs and 66 mRNAs. The expression of hsa_circRPRD1A (*P = 0.004*) and hsa_circHERPUD2 (*P = 0.003*) were significantly down-regulated in patients with coronary artery disease compared to controls. The area under the curve of hsa_circRPRD1A and hsa_circHERPUD2 is 0.689 and 0.662, respectively. Univariate and multivariable logistic regression analyses identified hsa_circRPRD1A (*OR = 0.613, 95%CI:0.380–0.987, P = 0.044*) as a protective factor for coronary artery disease. Based on the additive model, crossover analysis demonstrated that there was an antagonistic interaction between the expression of hsa_circHERPUD2 and alcohol consumption in subjects with coronary artery disease.

**Conclusion:**

Our findings imply that hsa_circRPRD1A and hsa_circHERPUD2 could be used as biomarkers for the diagnosis of coronary artery disease and provide epidemiological support for the interactions between circRNAs and classical coronary risk factors.

**Supplementary Information:**

The online version contains supplementary material available at 10.1186/s12920-023-01540-9.

## Introduction

Coronary artery disease (CAD), also known as ischemic heart disease, results from myocardial ischemia and hypoxia, which are associated with coronary artery atherosclerosis [1]. Myocardial infarction, arrhythmias, and even death can result from CAD if it is not diagnosed and treated in a timely manner. As stress myocardial perfusion imaging, coronary artery computed tomography angiography, and coronary angiography have developed, so has the diagnosis of CAD. However, in most cases, these examinations will not be considered, unless patients suffer from clinical symptoms. Unfortunately, approximately 1/4 of patients who experience myocardial infarction do so without any prior clinical symptoms [2]. Therefore, it is crucial to actively explore methods for early diagnosis of CAD.

Previous research has established that atherosclerosis is the result of plenty of complex factors, including the cells of the arterial wall, blood components, extracellular matrix, hemodynamic environment, immunity, and genetics [3]. Blood components such as monocytes, platelets, and low-density lipoprotein are all involved in the development of atherosclerosis. Peripheral blood mononuclear cells (PBMCs), as the name implies, are cells with a single nucleus in the peripheral blood that comprise both lymphocytes and monocytes. Monocytes play a critical role both in the development of atherosclerotic plaques and in the process of regression as members of the innate immune system [4]. Apart from that, a number of risk factors including age, blood pressure, blood lipids, smoking and obesity are also significant contributors to the onset of CAD [5, 6].

A unique form of non-coding RNA known as circular RNA (circRNA), which has a closed-loop structure and is not affected by RNA exonucleases [7]. Attempts have been made to explore the expression and function of circRNAs in CAD with microarray [8]. It should be noted, however, that such an exposition is unsatisfactory since microarray is not capable of recognizing novel circRNAs and only a limited number of circRNAs have been validated further. Research on circular RNAs has gradually intensified as high-throughput RNA sequencing (RNA-seq) and circular RNA-specific computational biology has been developed rapidly [9]. Therefore, we carried out a high-throughput RNA sequencing analysis in our prior study in order to determine the expression profile of circRNAs in CAD [10]. So far, however, there has been scanty discussion about the interaction effect of circRNAs and classical coronary risk factors on CAD.

Furthermore, this paper seeks to investigate the association between CAD and differentially expressed circRNAs in PBMCs. To achieve this purpose, a large cohort of individuals was analyzed using quantitative real-time polymerase chain reaction (qRT-PCR). Moreover, spearman correlation analysis, multivariable logistic regression analyses, as well as receiver operating characteristics analysis were conducted to evaluate the diagnostic value of circRNAs. In addition, the interaction effect of circRNAs and classical coronary risk factors is determined by crossover analysis. We will gain a better understanding of the pathogenesis of atherosclerosis by focusing on the association between circRNAs and CAD. In addition, it will also help us explore the interaction effect of circRNAs and classical coronary risk factors on CAD.

## Methods

### Study population

**Fig. 1:**
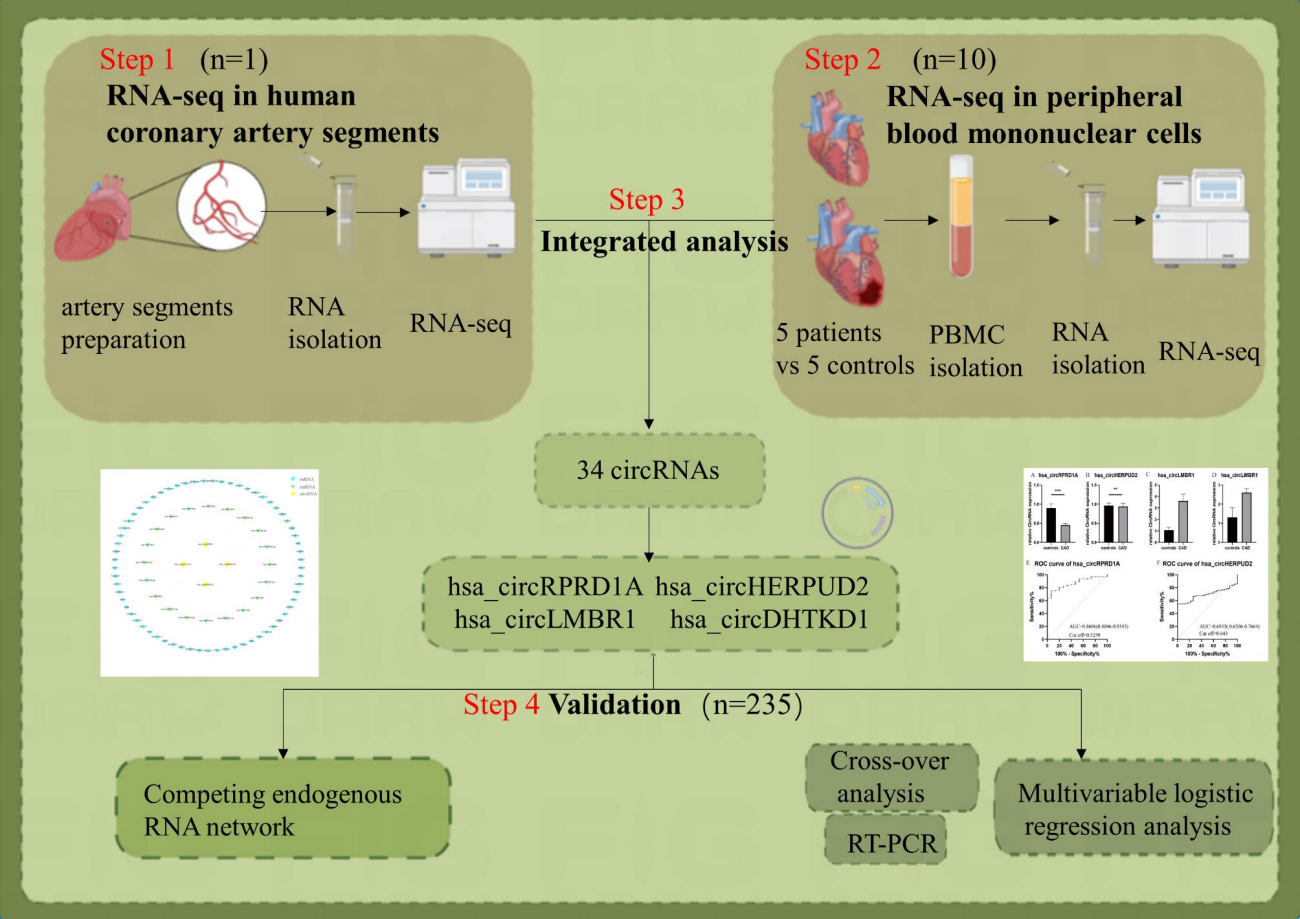
The flow chart of the present study. Integrated analysis between RNA sequencing results both in coronary artery segments and peripheral blood mononuclear cells was employed to figure out hub circRNA. 4 circRNAs was picked up by sanger sequencing from 34 circRNAs. Further investigation found out the expression of hsa_circRPRD1A and hsa_circHERPUD2 were significantly down-regulated in patients with CAD compared to controls, eventually

Our study population was divided into three groups in order to begin this process. Firstly, we obtained the epicardial coronary arteries from the autopsy specimen of an 81-year-old man who died from a heart attack. The autopsy was carried out by the Department of Human Anatomy in Nanjing Medical University. The preparation of coronary artery segments is described in detail in our previous study [11]. And then, Secondly, in order to investigate the relationship between circRNAs in PBMCs and CAD, transcriptome high throughput sequencing was conducted on 5 CAD patients and 5 healthy controls [12]. Finally, the validation group consisted of 256 individuals in the CAD group and 49 individuals in the control group. All patients were recruited at the First Affiliated Hospital of Nanjing Medical University. Figure 1 illustrates the flow chart of the present study.

Our study was carried out in accordance with the ethical guidelines of the First Affiliated Hospital of Nanjing Medical University’s ethical committee. We obtained written informed permission from all patients or their families according to the Declaration of Helsinki.

### Ascertainment of CAD

Whether coronary artery atherosclerosis occurs depends on the degree of luminal stenosis caused by arterial atherosclerosis. Coronary artery atherosclerosis is classified into four grades based on the cross-section of the stenosis that is the most severe: grade I, luminal stenosis area less than 25%; grade II, luminal stenosis area of 26–50%; grade III, 51–75%; and grade IV, 76–100% [13]. Generally, with the exception of coronary artery spasms, grade I-II atherosclerosis normally does not significantly reduce arterial blood flow or have a direct impact on CAD. Stenosis of grade III or above is closely related to CAD. Consequently, patients with at least one major coronary artery with stenosis greater than 50% were selected as the patient group, whereas individuals with all major coronary arteries with stenosis less than 50% were considered as the control group [14]. Patients with severe bacterial/viral/fungal infections, congenital heart abnormalities, cerebrovascular illnesses, cardiomyopathy, malignancy, severe hepatic and renal insufficiency, and a history of percutaneous coronary intervention were excluded from our study.

### Covariates

Baseline data including routine blood and blood biochemical tests were collected from the patient’s medical records. An interview-style questionnaire was used at baseline to collect information regarding smoking and alcohol consumption. Smoking is characterized as a person who has smoked continuously or cumulatively for 6 months or more in his lifetime. Further questions were asked about the frequency of their drinking (number of months, number of times per month), the type of alcoholic beverage they consumed (beer, liquor, wine, rice wine) and the amount of alcohol consumed per session. Lastly, individuals who consumed alcohol at least once a week during the past year and drank at least 12 g of alcohol per drink were considered to be alcohol consumers [15, 16]. The criteria for hypertension were an average systolic blood pressure greater than or equal to 140 mmHg, and/or diastolic blood pressure greater than or equal to 90 mmHg [17]. Diabetes is defined as fasting blood glucose exceeding than 7.8 mmol/l, or the use of hypoglycemic agents during the last 2 weeks [18]. Weight (kg) divided by height (m) squared was used to determine body mass index (BMI).

### Peripheral blood mononuclear cells collected

A fasting venous blood sample was obtained from each participant eight hours prior to coronary arteriography, which was pretreated with regular doses of aspirin and clopidogrel. To separate and preserve the lower blood cells, all samples were anticoagulated with ethylenediaminetetraacetic acid (EDTA)-2 K and centrifuged at 3000 rpm/centrifugation for 10 min. Then, we extracted the second white cloudy cell layer from it after centrifuging at 2000 rpm/centrifugation for 20 min with an equivalent lymphocyte separation medium (TBD, Tianjin, China). Finally, resuspended PBMCs with 1ml phosphate buffer saline (Biosharp, Anhui, China) and a counting was performed.

### RNA isolation

First, we extracted the total RNA from the PBMCs with TRIzol reagent (Invitrogen, Carlsbad, CA, USA). After PBMCs were lysed in TRIzol for ten minutes at 24 °C, 200 µl chloroform was added. Then, the resulting solution was gently mixed at room temperature for ten minutes and centrifuged with 12,000 g for twenty minutes at 4 °C. Next, we meticulously aspirated the upper aqueous phase into another centrifuge tube. We precipitated with equal isopropanol with centrifuged through 12,000 g×10 min, purified with 75% ethanol through centrifuged with 7500 g×5 min, and dissolved the sediment in RNase-free water. A NanoDrop 1000 spectrophotometer (Agilent Inc. USA) was used to measure RNA concentration and mass.

### Reverse transcription

To obtain complementary DNA (cDNA), we performed a reverse transcription reversed transcribe with RNA as the template in the presence of primers. Transcript one-step gDNA removal (YEASEN, Shanghai, China) was used to remove residual genomic DNA from the RNA template. And first-strand cDNA was synthesized using cDNA synthesis super-mix (YEASEN, Shanghai, China). Each stage is carried out on ice, and RNase contamination was strictly avoided throughout the procedure.

### Real-time polymerase chain reaction

The cDNA, SYBR q70P1CR master mix (EnzyArtisan, Shanghai, China), the primers, and diethyl pyrocarbonate treated water were configured according to the instructions. The aforementioned mixture was centrifuged at 3000 g for three minutes before performing qRT-PCR on StepOnePlus (Applied Biosystems, USA) equipment. Each well was repeated for three times to ensure the quantification accuracy. The expression of the circRNA was calculated using the 2^−ΔΔCt^ method, and GAPDH was employed as an internal reference gene.

### Bioinformatic analyses

The microRNAs that may be in competition with our four targeted circRNAs were obtained using miRanda-3.3a [19]. We constructed a competing endogenous RNA network by predicting the target genes of the top 5 miRNAs ranked by their structure score of every circRNAs through TargetScan7.0 [20] Cytoscape 3.8.2 was used to visualized the related lncRNA, miRNA and miRNA target genes [21].

### Statistical analysis

The data were analyzed by SPSS (version26.0, USA) and GraphPad Prism (version8.0, USA). Normally distributed data were presented by mean ± standard deviation (SD) determined by t-test, otherwise presented as median (interquartile range) and determined by the Wilcoxon-Mann-Whitney test. The Spearman correlation and multivariate regression were used to further identify the relationship between circRNAs expression levels and clinical characteristics in CAD patients. The receiver operating characteristic (ROC) curve analysis was carried out to evaluate the potential diagnostic value of circRNAs. Multivariate logistic regression and unconditional logistic regression were used to evaluate relationships between relative expression levels of circRNAs and CAD. We conducted crossover analyses to identify the interaction of circRNAs with classical coronary risk factors in subjects with CAD [22]. The additive interaction was evaluated using the relative excess risk owing to interaction (RERI), attributable proportion of interaction (AP), and synergy index (S). There was no additive interaction when the 95% confidence interval(95%*CI*) of RERI contained 0 or 95%*CI* of S contained 1, which demonstrate that the combined effect of circRNA and classical coronary risk factors is equal to the sum of their individual effects. For further analysis, we used one-way analysis of variance (ANOVA) and post-hoc tests to assess differences between groups. An analysis of normality and homogeneity of variance of the data was conducted using the Levene test and the Brown-Forsythe test. *P < 0.0*5 was considered to be statistically significant.

## Result

### Overview of RNA sequencing results in PBMCs

**Fig. 2:**
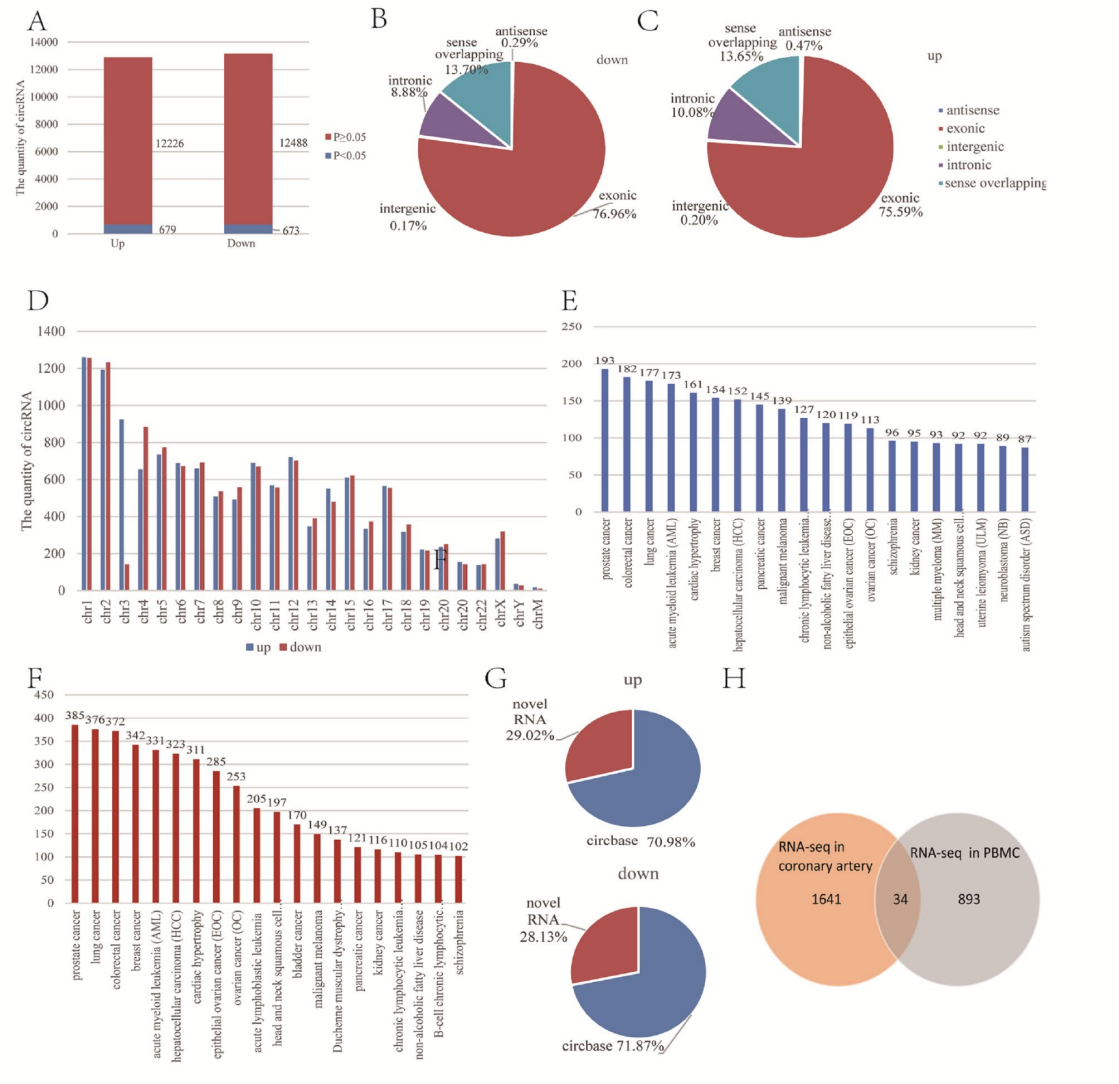
Overview of circRNAs RNA sequence results in peripheral blood mononuclear cells. (A) Proportion exhibiting the number of circRNAs in the up-regulation group and down-regulation group. CircRNA with a significant difference is shown in the blue and others in the red. (B-C) Pie charts exhibit the catalog of the circRNA, including antisense, exonic, intergenic, intronic, and sense overlapping in the two groups. (D) Chromosomal distribution of all identified circRNAs. (E-F) Histogram displaying the top 20 circRNA-associated diseases of all identified circRNA indicated by circ2Trait in the up-regulation group and down-regulation group, respectively. (G) Proportion of novel circRNAs in the two groups. (H) Venn diagram exhibiting number of common circRNAs between RNA-seq in coronary artery and PBMCs.

The relative expression level of circRNA in CAD patients in PBMCs was determined by RNA sequencing [10]. Compared with normal controls, the expression levels of 12,905 circRNAs and 13,161 circRNAs was up-regulated and down-regulated in the CAD group, respectively **(**Fig. 2A**)**. What’s more, there are 679 circRNAs and 673 circRNAs were assessed to be significantly different expressions between the two groups (*P < 0.05*). At the same time, our results revealed approximately 75% of circRNAs were exonic. The proportion was generally 13% at sense overlapping, approximately 10% at intronic, and less than 1% of circRNAs were classified as antisense and intergenic **(**Fig. 2B-C**)**. Besides, the circRNAs locations were identified on all human chromosomes and one notable exception was that there are 29 circRNAs positioned in chrM **(**Fig. 2D**)**.

In addition, circRNA-associated diseases were categorized by circ2Trait (http://gyanxet-beta.com/circdb/) [23]. There are 5011 up-regulated circRNAs linked to 86 different kinds of diseases and 4836 down-regulated circRNAs associated with 93 different kinds of diseases **(**Fig. 2E-F**)**. Among these circRNAs, 472 circRNAs were correlated with cardiac hypertrophy. Furthermore, the results described 10,426 novel circRNAs not been mentioned in any circRNA database before **(**Fig. 2G**)**.

### Transcriptome profile of human coronary arteries by RNA-Seq

This research is a component of our series whose findings have already been reported [24]. CircRNAs expression was statistically calculated and normalized by TPM [25].

### Integrated RNA-seq analysis

**Fig. 3:**
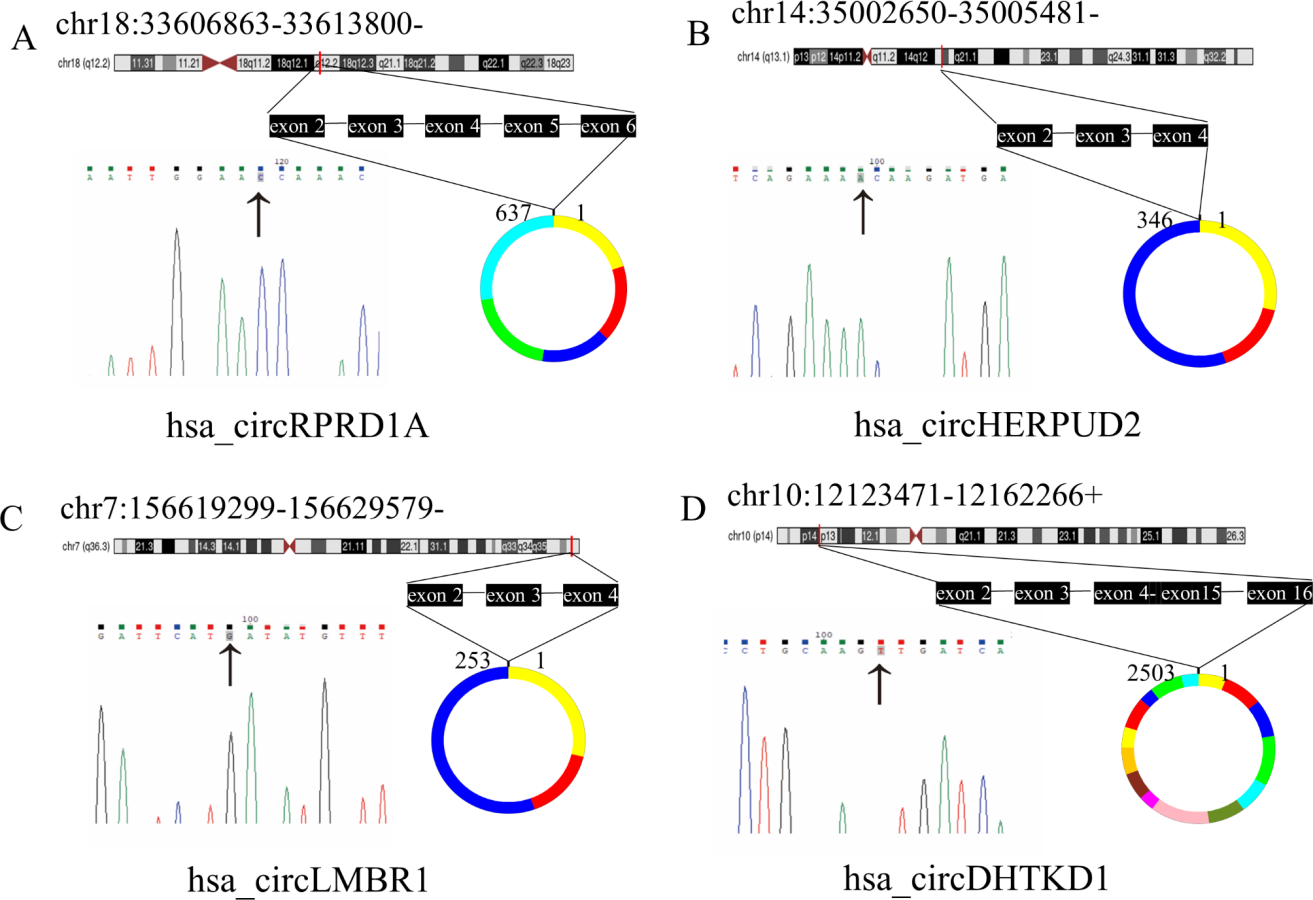
Sanger sequencing of selected candidate circRNAs. The black arrow points to the back-splice junctions. The number above the ring indicates how many bases the circRNA is made up of and every color represents an exon, respectively

In our previous study, the circRNA profiles in PBMCs of CAD patients were identified by RNA-seq. We also characterized human coronary artery transcriptomes by RNA-seq at the same time. A total of 34 circRNAs were not only detected in coronary artery segments but also differently expressed in PBMCs of CAD patients (fold-change ≥ 2 and *P < 0.05*) **(**Fig. 2H**)**. The characteristics of the circRNAs mentioned above are displayed in Table 1. Additionally, the expression levels of 34 circRNAs in human coronary artery segments are listed in Table 2. Then, we designed primers for circRNA junction sites. The qRT-PCR products were sequenced in order to determine the specificity of the primers used for amplification, and the results were compared with the reference sequence found in the circBase database. The Sanger sequencing results revealed that the product sequences were compatible with the database sequence, indicating that hsa_circRPRD1A, hsa_circHERPUD2, hsa_circLMBR1, and hsa_circDHTKD1 could be amplified specifically by qRT-PCR **(**Fig. 3**).**

**Table 1:** The characteristics of the 34 selected circRNAs

GeneName	CircRNAID	logFC	best_transcript	Catalog
hsa_circKPNB1	chr17:45741525–45,752,148+	3.201184	NM_002265	exonic
hsa_circRPRD1A	chr18:33606863-33613800-	1.998969	NM_018170	exonic
hsa_circSETBP1	chr18:42281140–42,292,759+	-3.64891	NM_015559	sense overlapping
hsa_circFKBP8	chr19:18650181-18650530-	-3.2811	NM_012181	exonic
hsa_circHERPUD2	chr7:35707044-35712888-	-1.10267	NM_022373	exonic
hsa_circLMTK2	chr7:97820040–97,823,884+	-1.78242	NM_014916	exonic
hsa_circSHQ1	chr3:72881520-72893574-	3.739837	NM_018130	exonic
hsa_circMRRF	chr9:125042722–125,054,119+	-2.92626	NM_138777	exonic
hsa_circPER2	chr2:239184384-239186596-	-3.0208	NM_022817	exonic
hsa_circFOCAD	chr9:20819795–20,823,114+	-1.43195	ENST00000338382	exonic
hsa_circUTRN	chr6:144808684–144,832,259+	-3.83134	NM_007124	exonic
hsa_circSEMA3C	chr7:80418622-80440017-	2.501538	NM_006379	exonic
hsa_circMAPK1	chr22:22153301-22162135-	-2.51592	NM_002745	exonic
hsa_circLMBR1	chr7:156619299-156629579-	-1.10466	NM_022458	exonic
hsa_circAMD1	chr6:111208708–111,211,559+	-1.52343	NM_001634	exonic
hsa_circHECTD2	chr10:93185038–93,221,941+	-2.73039	NR_104291	exonic
hsa_circKMT2E	chr7:104678573–104,681,470+	-2.27635	NM_182931	exonic
hsa_circDHTKD1	chr10:12123471–12,162,266+	-2.47823	NM_018706	exonic
hsa_circPTPN13	chr4:87685746–87,689,129+	-1.82586	NM_006264	exonic
hsa_circSTX12	chr1:28116073–28,120,143+	-1.04929	NM_177424	exonic
hsa_circGPBP1L1	chr1:46105882-46108171-	1.701038	NM_021639	exonic
hsa_circTPP2	chr13:103275227–103,280,274+	-2.14369	NM_003291	exonic
hsa_circSLC19A2	chr1:169446393-169446995-	3.12843	NM_006996	exonic
hsa_circCNST	chr1:246754814–246,755,243+	-1.22787	NM_152609	exonic
hsa_circPIWIL4	chr11:94316614–94,341,852+	3.489148	NM_152431	exonic
hsa_circLARP4	chr12:50821545–50,855,130+	3.195552	NM_052879	exonic
hsa_circPOLD1	chr19:50902108–50,902,741+	-3.54791	NM_002691	exonic
hsa_circGFPT1	chr2:69575303-69590802-	-2.94654	NM_002056	exonic
hsa_circNBEAL1	chr2:203974873–203,991,649+	3.7599	NM_001114132	exonic
hsa_circCLASP2	chr3:33661095-33686395-	-3.31589	NM_015097	exonic
hsa_circTBCK	chr4:107114766-107133992-	3.483071	NM_001290768	exonic
hsa_circDDHD2	chr8:38105231–38,111,236+	-3.64212	NM_015214	exonic
hsa_circPSMD5	chr9:123593609-123595734-	-2.10354	NM_005047	exonic

CircRNAID, the ID of the identified circRNA by DCC. logFC: The value of the multiplicative change between the two groups which transformed by log2, the circRNAs are up-regulated in CAD group when logFC > 0, otherwise, down-regulated. GeneName, the name of the circRNA-associated gene. Catalog: the catalog of the circRNA.

**Table 2:** List of 34 cirRNAs that were co-expressed in the coronary artery segments and PBMCs of CAD patients

circRNAs	LM.tpm	RCA-D.tpm	LAD-D.tpm	LCX-P.tpm	LCX-M.tpm	LAD-P.tpm	LAD-M.tpm	RCA-M.tpm
hsa_circKPNB1	0.000	0.000	0.000	0.000	0.000	0.000	338.753	0.000
hsa_circRPRD1A	1213.040	1425.794	390.320	961.406	1405.191	445.991	1287.263	1275.917
hsa_circSHQ1	0.000	0.000	0.000	412.031	0.000	0.000	0.000	0.000
hsa_circSEMA3C	454.890	0.000	195.160	206.016	0.000	267.594	135.501	159.490
hsa_circGPBP1L1	0.000	0.000	487.900	0.000	247.975	267.594	135.501	0.000
hsa_circSLC19A2	0.000	259.235	195.160	0.000	0.000	0.000	338.753	0.000
hsa_circPIWIL4	0.000	0.000	0.000	0.000	413.292	0.000	0.000	0.000
hsa_circLARP4	0.000	0.000	0.000	137.344	0.000	0.000	0.000	398.724
hsa_circNBEAL1	0.000	0.000	0.000	549.375	0.000	267.594	271.003	398.724
hsa_circTBCK	0.000	518.471	0.000	137.344	0.000	267.594	203.252	159.490
hsa_circSETBP1	151.630	0.000	0.000	137.344	495.950	0.000	0.000	0.000
hsa_circFKBP8	0.000	1166.559	683.060	961.406	1570.508	356.792	271.003	558.214
hsa_circHERPUD2	682.335	0.000	292.740	412.031	0.000	624.387	338.753	318.979
hsa_circLMTK2	606.520	907.323	683.060	549.375	495.950	445.991	609.756	478.469
hsa_circMRRF	303.260	0.000	683.060	480.703	0.000	178.396	0.000	318.979
hsa_circFOCAD	530.705	0.000	0.000	0.000	247.975	267.594	135.501	0.000
hsa_circPTPN12	758.150	1555.412	683.060	412.031	826.583	356.792	474.255	398.724
hsa_circPER2	0.000	648.088	0.000	206.016	0.000	0.000	0.000	0.000
hsa_circUTRN	379.075	0.000	0.000	0.000	0.000	0.000	0.000	0.000
hsa_circMAPK1	454.890	259.235	292.740	412.031	165.317	0.000	0.000	0.000
hsa_circLMBR1	909.780	518.471	1170.960	480.703	247.975	535.189	203.252	558.214
hsa_circAMD1	530.705	777.706	585.480	274.688	826.583	981.179	745.258	956.938
hsa_circHECTD2	227.445	518.471	0.000	343.359	247.975	356.792	271.003	398.724
hsa_circKMT2E	606.520	648.088	683.060	412.031	495.950	267.594	542.005	797.448
hsa_circDHTKD1	0.000	0.000	0.000	274.688	0.000	178.396	135.501	398.724
hsa_circPTPN13	758.150	388.853	780.640	1030.078	578.608	802.783	1084.011	1435.407
hsa_circSTX12	0.000	0.000	0.000	0.000	0.000	0.000	0.000	0.000
hsa_circTPP2	303.260	0.000	292.740	961.406	578.608	178.396	271.003	318.979
hsa_circCNST	151.630	0.000	0.000	274.688	495.950	445.991	0.000	0.000
hsa_circPOLD1	682.335	1036.941	975.800	412.031	578.608	0.000	745.258	318.979
hsa_circGFPT1	0.000	0.000	0.000	0.000	0.000	0.000	135.501	0.000
hsa_circCLASP2	0.000	259.235	195.160	412.031	495.950	0.000	271.003	318.979
hsa_circDDHD2	151.630	0.000	0.000	0.000	165.317	178.396	338.753	0.000
hsa_circPSMD5	379.075	0.000	292.740	961.406	743.925	356.792	406.504	1036.683

LM, left main trunk; RCA-d, distal segment of the right coronary artery LAD-d: distal segment of the left anterior descending; LCX-p, proximal segment of the left circumflex; LCX-m, midsegment of the left circumflex; LCX-d, distal segment of the left circumflex; LAD-P, proximal segment of the left anterior descending; LAD-m, midsegment of the left anterior descending; RCA-m, midsegment of the right coronary artery

### Construction of competing endogenous RNA networks

**Fig. 4:**
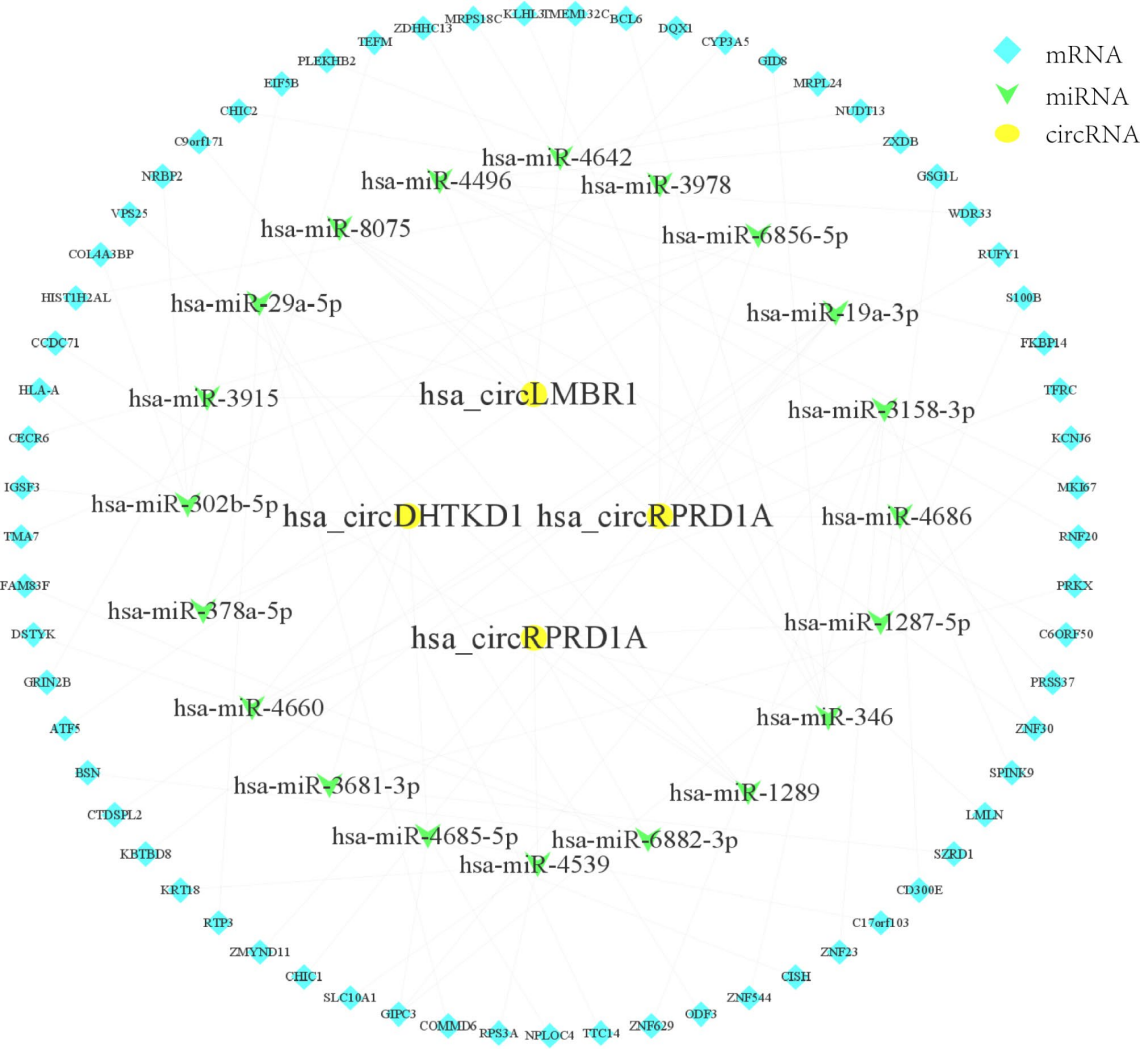
The circRNA-miRNA-mRNA interaction network. Yellow round nodes indicate four selected circRNAs. The green arrow and the blue diamond represent miRNAs and mRNAs, respectively. The lines between them indicate their interconnectedness

It is known that circRNA regulates gene expression by competitively binding microRNAs [26, 27]. Firstly, we predicted the potential binding miRNAs of hsa_circRPRD1A, hsa_circHERPUD2, hsa_circLMBR1, and hsa_circDHTKD1 by miRanda. Our findings revealed interactions between 228 miRNAs and 4 circRNAs. And then target gene prediction was performed on the top 20 miRNAs ranked by structure score, and these target genes were analyzed jointly with the mRNAs in PBMCs from CAD patients in our previous studies, and 66 target mRNAs were obtained that may be involved in the development of CAD under circRNAs regulation. As shown in Fig. 4, the rank top 5 miRNAs and the related 66 mRNAs were used to construct a circRNA-miRNA-mRNA network.

### Validation of the circRNA expression

**Fig. 5:**
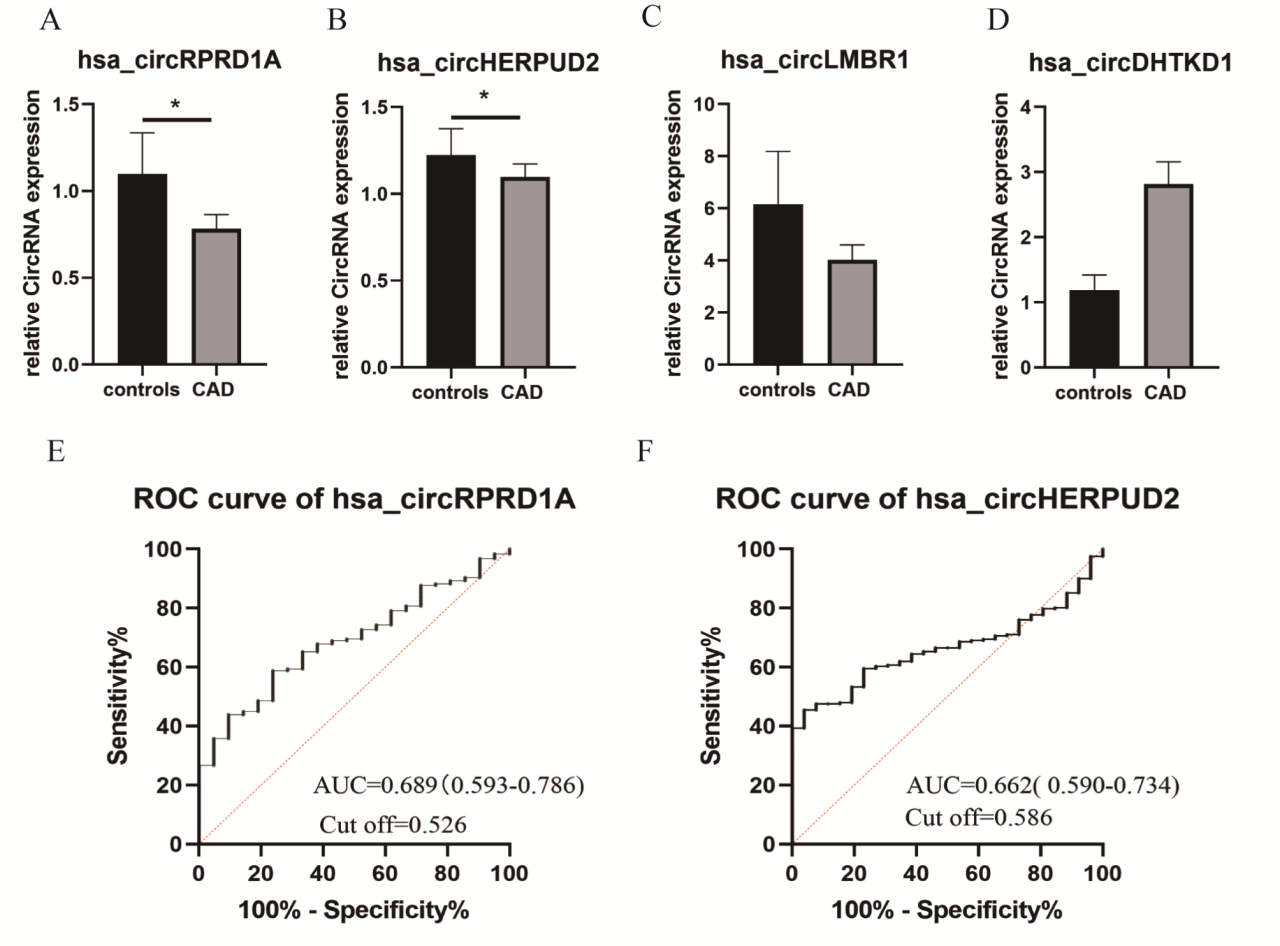
Validation of the circRNAs expression and identification the potential biomarker (A-D) qRT-PCR analysis of expression of 4 circRNAs in a large sample of CAD patients and healthy controls. *P < 0.05, **P < 0.001. (E-F) ROC curve analysis of hsa_circRPRD1A and hsa_circHERPUD2 discrimination of CAD patients from healthy controls

The quantitative real-time polymerase chain reaction was employed to validate the expression level of hsa_circRPRD1A, hsa_circHERPUD2, hsa_circLMBR1, hsa_circDHTKD1 in a large cohort. First, qRT-PCR primers for circRNA were listed in supplementary Table 1. Second, 256 CAD patients and 49 healthy controls defined by coronary angiography were included in this study. The summary statistics of the clinical characteristics are depicted in Table 3. Gender (*p < 0.001)*, cardiac troponin T (*P = 0.031*), and Gensini score (*p < 0.0001*) were significantly different between the two groups. Finally, the relative expression levels of the four circRNAs were evaluated in PBMCs. Compared with controls, the expression levels of hsa_circRPRD1A (*P = 0.004*) and hsa_circHERPUD2 (*P = 0.003*) were significantly decreased in CAD patients. In contrast to the controls, the expression levels of hsa_circLMBR1 *(P = 0.906)*, and hsa_circDHTKD1 *(P = 0.288)* presented were up-regulated in the CAD group. However, no significant difference between the two groups was evident **(**Fig. 5A-D**)**.

**Table 3:** Clinical characteristics of study subjects between CAD and control

Variables	CAD patient(n = 256)	Control(n = 49)	*Z/t*	*P value*
Gender(M/F)	189/67	23/26	-3.740	< 0.001**
Age (years)	66.000(57.000-72.750)	65.000(54.750–72.250)	-0.720	0.471
BMI (kg/m2)	25.028 ± 3.512	24.988 ± 3.599	-0.065	0.831
Smoking, n (%)	41.3	35.6	-0.720	0.471
Drinking, n (%)	28.9	17.8	-1.535	0.125
Hypertension, n (%)	63.0	48.9	-1.781	0.075
Diabetes mellitus, n (%)	20.4	11.1	-1.460	0.144
SBP (mmHg)	130.317 ± 17.282	131.867 ± 18.910	0.512	0.536
DBP (mmHg)	75.000(69.000–84.000)	78.000 (72.500–87.000)	-1.780	0.075
Total cholesterol(mmol/L)	3.605(3.065–4.373)	4.075(3.125–4.665)	-1.444	0.149
TG (mmol/L)	1.270(0.948–1.713)	1.205(0.880–1.635)	-0.331	0.741
HDL-C (mmol/L)	0.980(0.860–1.135)	1.085(0.886–1.286)	-2.056	0.040*
LDL-C (mmol/L)	2.110(1.728–2.705)	2.230(1.766–2.843)	-0.881	0.378
GLU (mmol/L)	5.035(4.518–5.853)	4.980(4.486–5.650)	-0.777	0.437
Lp (a) (mg/L)	172.000(84.000-382.000)	131.500(61.750–320.000)	-1.110	0.267
CKMB (ug/dl)	2.320(1.780–3.150)	2.330(1.5075–2.998)	-0.711	0.477
cTnT(ng/dl)	11.265(6.983–24.323)	9.400(5.510–20.470)	-2.154	0.031 *
EF value (%)	62.700(60.700–64.700)	63.000(61.400-64.725)	-0.884	0.377
Gensini score	48.000(24.000-93.750)	0.000(0.000-4.500)	-10.824	< 0.001**
hsa_circRPRD1A	0.466(0.215–0.728)	0.613(0.490–1.465)	-2.843	0.004*
hsa_circHERPUD2	0.722(0.402–1.633)	0.976(0.765–1.448)	-2.165	**0.003** ^*^
hsa_circLMBR1	1.520(0.582–4.384)	1.286(0.569–4.746)	-0.118	0.906
hsa_circDHTKD1	1.318(0.711–3.988)	1.062(0.570–1.513)	-1.062	0.288

^*^P < 0.05, ^**^P < 0.001. BMI: body mass index, SBP: systolic blood pressure, DBP: diastolic blood pressure, TG: triglyceride, HDL-C high-density lipoprotein cholesterol, LDL-C: low-density lipoprotein cholesterol, GLU: fasting blood glucose, Lip (a): lipoprotein (a), CKMB: creatine kinase-myocardial band, EF: ejection fraction. cTnT: cardiac troponin T. Normally distributed presented by Mean ± SD determined by t-test, the skewness distribution parameters were presented as median (interquartile range) and determined by the Wilcoxon-Mann-Whitney test

### Identification of potential circRNA biomarkers

The spearman correlation analysis displayed that the expression level of hsa_circHERPUD2 was positively correlated with BMI (*r = 0.152, P = 0.026*) and the expression level of hsa_circLMBR1 was positively correlated with high-density lipoprotein cholesterol (*r = 0.212, p < 0.001*). Besides, it can be seen from Table 4 that the expression level of hsa_circRPRD1A was positively correlated with hsa_circHERPUD2 expression (*r = 0.394, p < 0.001*) and hsa_circDHTKD1 (r = 0.156, *P = 0.046*). Additionally, the expression level of hsa_circLMBR1 was also positively correlated with hsa_circDHTKD1 (*r = 0.564, p < 0.001*). After adjusting for a number of different clinical parameters in the multivariate linear regression model, we discovered that lip(a) (*β = 0.390 P = 0.008*), high-density lipoprotein cholesterol (*β = 0.212, P = 0.001*), and lipoprotein (a) (*β = 0.134, P = 0.040)* were independent factors associated with the expressions of hsa_circRPRD1A, hsa_circHERPUD2, and hsa_circLMBR1, respectively.

**Table 4:** Correlation between baseline characteristic and circRNAs expression level in CAD patient group

Parameters	hsa_circRPRD1A				hsa_circHERPUD2				hsa_circLMBR1				hsa_circDHTKD1			
	r	*p*	β	*p*	r	*p*	β	*p*	r	*p*	β	*p*	r	*p*	β	*p*
Age (years)	-0.077	0.252	0.168	0.343	-0.025	0.699	**-0.327**	0.043*	-0.062	0.348	0.209	0.164	-0.035	0.622	**-0.298**	0.034*
BMI (kg/m2)	0.078	0.291	0.001	0.997	**0.152**	0.026*	0.217	0.077	-0.087	0.215	-0.032	0.782	**0.205**	0.006*	-0.056	0.603
SBP (mmHg)	-0.02	0.778	-0.070	0.660	-0.004	0.948	-0.061	0.678	-0.061	0.365	-0.115	0.395	-0.023	0.755	**0.268**	0.032*
DBP (mmHg)	0.035	0.623	0.115	0.516	-0.026	0.697	-0.141	0.391	-0.063	0.349	0.128	0.399	-0.051	0.479	-0.275	0.050
Total cholesterol (mmol/L)	-0.019	0.787	1.398	0.307	-0.016	0.809	-1.711	0.175	-0.024	0.722	-1.199	0.304	0.045	0.526	1.562	0.154
TG (mmol/L)	-0.029	0.682	-0.127	0.467	-0.024	0.713	0.205	0.203	-0.042	0.531	0.127	0.392	-0.003	0.969	-0.121	0.391
HDL-C (mmol/L)	-0.035	0.622	-0.280	0.330	0.041	0.529	0.388	0.142	**0.212**	0.001*	0.197	0.422	-0.037	0.601	-0.151	0.516
LDL-C (mmol/L)	0.053	0.445	-1.292	0.293	0.032	0.618	1.557	0.169	-0.069	0.299	1.016	0.332	0.098	0.167	-1.413	0.151
GLU (mmol/L)	-0.02	0.773	-0.185	0.176	**0.185**	0.004*	0.092	0.469	-0.041	0.542	0.054	0.643	0.094	0.186	-0.140	0.205
Lp (a) (mg/L)	-0.051	0.469	**0.390**	0.008*	-0.020	0.757	0.058	0.682	**0.134**	0.040*	-0.211	0.097	0.082	0.239	0.154	0.203
Gensini score	**0.158**	0.023*	-0.151	0.308	-0.050	0.439	0.227	0.096	0.113	0.088	0.220	0.078	0.042	0.555	**-0.242**	0.040*
EF (%)	-0.014	0.873	-0.095	0.478	0.011	0.884	-0.065	0.599	0.066	0.422	-0.009	0.939	0.032	0.709	0.001	0.995
hsa_circRPRD1A	NA	NA	NA	NA	0.394	< 0.001	0.085	0.514	0.139	0.069	0.094	0.429	0.156	0.046	-0.079	0.487
hsa_circHERPUD2	**0.394**	**< 0.001** **	0.099	0.514	NA	NA	NA	NA	0.061	0.399	0.100	0.441	0.070	0.352	0.090	0.462
hsa_circLMBR1	0.139	0.069	0.130	0.429	0.061	0.399	0.117	0.441	NA	NA	NA	NA	0.115	0.118	**0.564**	< 0.001**
hsa_circDHTKD1	**0.156**	0.046*	0.121	0.487	0.070	0.352	0.119	0.462	0.115	0.118	**0.631**	< 0.001**	NA	NA	NA	NA

* P < 0.05, ** P < 0.001.BMI: body mass index, SBP: systolic blood pressure, DBP: diastolic blood pressure, CRP: C-reaction protein, TG: triglyceride, HDL-C high-density lipoprotein cholesterol, LDL-C: low-density lipoprotein cholesterol, GLU: fasting blood glucose, Lip (a): lipoprotein (a), EF: ejection fraction.

### Diagnostic values of circRNA expressions

The results obtained from the ROC analysis of hsa_circRPRD1A and hsa_circHERPUD2 are presented in Fig. 5E-F. The area under the ROC curve (AUC) of hsa_circRPRD1A was 0.689 (95% *CI*:0.593–0.786, *P = 0.004*). A hsa_circRPRD1A expression level of 0.526 was the most appropriate cut-off value with sensitivity and specificity of 0.588 and 0.782, respectively. As for hsa_circHERPUD2, the AUC was 0.662 (95% *CI*:0.590–0.734, *P = 0.006*), and the cut-off values was 0.662 of 0.479 and 0.808 for sensitivity and specificity, respectively.

### Univariate and multivariable logistic regression analysis

Multivariable logistic regression analyses were conducted to investigate risk factors and predict CAD probability. As shown in Table 5, without adjustment, hsa_circRPRD1A acts as a protective factor for coronary artery disease with an odds ratio of 0.625 (95% *CI*: 0.412–0.948, *P = 0.027*). After age, gender, BMI, smoking, alcohol consumption, diabetes mellitus, hypertension, TC, TG, Apo (a), fasting blood glucose levels, and LDL-C were adjusted, the expression of hsa_circRPRD1A (*OR = 0.613, 95%CI:0.380–0.987, P = 0.044*) remained significantly associated with CAD prevalence. Unfortunately, no evidence was found for multivariable logistic associations between hsa_circHERPUD2 (*P = 0.837*), hsa_circLMBR1 (*P = 0.229*), hsa_circDHTK-D1 (*P = 0.441*) and CAD.

**Table 5:** Univariate and multivariable logistic regression analyses of the circRNAs and environmental factors in CAD risk

Factors	Univariate analysis		Model 1		Model 2	
	*OR* (95% *CI*)	*p*	*OR* (95% *CI*)	*p*	*OR* (95% *CI*)	*p*
hsa_circRPRD1A	0.625(0.412–0.948)	**0.027**	0.628(0.404-0977)	**0.039**	0.613(0.380–0.987)	**0.044**
hsa_circHERPUD2	0.965(0.686–1.356)	0.837	0.946(0.640–1.399)	0.783	0.883(0.578–1.348)	0.565
hsa_circLMBR1	0.979(0.945–1.014)	0.229	0.983(0.947–1.021)	0.384	0.989(0.949–1.030)	0.989
hsa_circDHTKD1	1.069(0.902–1.266)	0.441	1.072(0.887–1.296)	0.471	1.096(0.891–1.348)	0.384

Multivariate logistic regression Model 1: Adjusted for age, gender, BMI, smoking, drinking, diabetes mellitus and hypertension

Multivariate logistic regression Model 2: Adjusted for age, gender, BMI, smoking, drinking, diabetes mellitus, hypertension, TC, TG, Apo(a), and LDL-C.

*OR*: odds ratio, *CI*: confidence interval, TC: Total cholesterol, TG: triglyceride, Apo(a): apolipoprotein a, LDL-C: low-density lipoprotein cholesterol

### Analysis of variance

The study population was classified into mild, ordinary, severe and critical groups according to the Gensini score quartile. The ANONA found no significant differences in the expression of hsa_circRPRD1A, hsa_circHERPUD2, and hsa_circLMBR1 between cases with different disease severity (Table 6**)**. Interestingly, there were a significant difference in the expression of hsa_circDHTKD1 between the four groups (F = 3.739, *P = 0.012*). What’s more, the Bonferroni test was used for multiple comparisons and revealed that the expression of hsa_circDHTKD1 between ordinary cases and severe cases was significantly different (*P = 0.011*).

**Table 6:** One-way analysis of variance in the study population categorized by Gensini scores quartile

CircRNA	Mild cases (n = 76)	Ordinary cases (n = 78)	Severe cases (n = 76)	critical cases (n = 75)	F value	*p-value*
hsa_circRPRD1A	0.997 ± 1.009	0.710 ± 0.790	0.577 ± 0.729	0.614 ± 0.752	2.388	0.070
hsa_circHERPUD2	1.337 ± 1.139	1.163 ± 1.084	1.044 ± 1.010	1.085 ± 1.025	0.785	0.504
hsa_circLMBR1	2.124 ± 2.402	2.880 ± 2.725	2.210 ± 2.531	1.852 ± 2.106	1.731	0.162
hsa_circDHTKD1	1.917 ± 2.442	3.104 ± 2.517* ^**OS**^	1.726 ± 1.867	2.163 ± 1.867	3.739	**0.012**

For the overall analysis, a one-way ANOVA was used, while for the multiple comparisons, the Bonferroni test was used. The significance marker *^OS^ represents that there was a significant difference between Ordinary cases and Severe cases (*p < 0.05*)

### Crossover analysis of circRNA and risk factors

Based on the additive model, we analyzed the modification effect interaction between the expression of circRNA and CAD risk factors. As can be seen from Table 7, the expression of hsa_circRPRD1A is negatively interacted with smoking (S = 0.658, API = 2.395, RERI = 0.428) and hypertension (S=-2.117, API=-3.182, RERI=-1.007) in patients with CAD compared to controls. Due to the 95%*CI* of S containing 1, the interaction between hsa_circRPRD1A and smoking or hypertension was not considered significant. Simultaneously, the expression of hsa_circHERPUD2 negatively interacted with smoking (S = 0.682), drinking (S = 0.500), and hypertension (S=-0.130). Alcohol consumption was the only variable that significantly negative interacted with the expression of hsa_circHERPUD2 (API = 4.415, RERI = 0.707), suggesting that there was an antagonistic interaction between the expression of hsa_circHERPUD2 and alcohol consumption in subjects with CAD. Based on a further analysis of alcohol consumption, it was revealed that patients who consumed alcohol had higher levels of high-density lipoprotein cholesterol than those who did not consume alcohol (Supplementary Table 2).

**Table 7:** Interaction between circRNA and classical coronary risk factors of CAD.

Risk factors		CAD	Control	*OR* (95%*CI*)	p-value	Risk factors		CAD		Control		*OR* (95%*CI*)		p-value
hsa_circRPRD1A						hsa_circHERPUD2								
Smoking						Smoking								
-	-	65	2	1.000		-	-	56		2				
+	-	43	11	0.631(0.085–4.654)	0.651	+	-	66		13		0.696(0.094–5.157)		0.723
-	+	41	2	0.120(0.025–0.570)	0.008	-	+	39		2		0.181(0.039–0.838)		0.029
+	+	29	5	0.178(0.033–0.974)	**0.047**	+	+	46		7		0.235(0.046–1.185)		0.079
S (95%*CI*) = 0.658(-0.727-2.042) API (95%*CI*) = 2.395(-0.834-5.624) RERI (95%*CI*) = 0.428(-1.210-2.065)						S (95%*CI*) = 0.682(-1.111-2.474) API (95%*CI*) = 1.521(-0.405-3.447) RERI (95%*CI*) = 0.357(-1.555-2.270)								
Drinking						Drinking								
-	-	79	3	1.000		-	-	66		2				
+	-	51	13	1.025(0.102–10.278)	0.983	+	-	82		17		0.439(0.059–3.273)		0.422
-	+	27	1	0.149(0.040–0.549)	0.004	-	+	29		2		0.146(0.033–0.655)		0.012
+	+	20	3	0.253(0.047–1.350)	0.108	+	+	29		3		0.293(0.046–1.848)		0.191
S (95%*CI*) = 0.905(-2.546-4.355) API (95%*CI*) = 0.311(-0.605-1.228) RERI (95%*CI*) = 0.079(-2.752-2.910)						**S (95%** ***CI*** **) = 0.500(0.218–0.782) API (95%** ***CI*** **) = 4.415(1.230–7.599)** **RERI (95%** ***CI*** **) = 0.707(0.315–1.099)**								
Hypertension						Hypertension								
-	-	34	2			-	-	35		3				
+	-	28	8	2.118(0.286–15.678)	0.463	+	-	42		9		5.143(0.515–51.36)		0.163
-	+	72	2	0.206(0.040–1.049)	0.057	-	+	60		1		0.400(0.100-1.592)		0.194
+	+	43	8	0.316(0.063–1.587)	0.162	+	+	69		11		0.538(0.141–2.053)		0.364
S (95%*CI*) =-2.117(-16.124-11.890) API (95%*CI*) = -3.182(-9.144-2.779) RERI (95%*CI*) =-1.007(-5.935-3.922)						S (95%*CI*) =-0.130(-3.330-3.069) API (95%*CI*) =-7.457(-19.505-4.590) RERI (95%*CI*) =-4.008(-17.065-9.050)								

*OR*: odds ratio, *CI*: confidence interval, S: the synergy index, API: attributable proportions of interaction, RERI: lative excess risk of interaction, HDL-C high-density lipoprotein cholesterol, LDL-C: low-density lipoprotein cholesterol

## Discussion

We investigated whether circRNAs have affect atherosclerosis pathogenesis and explored biomarkers for the diagnosis of CAD in our present study. To examine the relationship between differently expressed circRNAs in PBMCs with CAD, we performed qRT-PCR in large cohort populations and conducted statistical analysis. According to our results, firstly, we verified the specificity of the amplification primers of hsa_circRPRD1A, hsa_circHERPUD2, hsa_circLMBR1, and hsa_circDHTKD1 by sanger sequencing. Secondly, we discovered that the expression level of hsa_circRPRD1A and hsa_circHERPUD2 were significantly down-regulated in CAD patients compared with controls. Last but not least, ROC curve analysis, multivariable logistic regression analysis along with crossover analysis observed that hsa_circRPRD1A and hsa_circHERPUD2 can function as diagnostic biomarkers and together with disease risk factors play a crucial role in the development of CAD.

CircRNAs have been discovered to play a role in the pathological of cardiovascular processes, such as myocardial infarction [28], heart failure [29], and overall atherosclerosis [30]. In addition, PBMCs are a key player in atherosclerosis plaque development. The impact of circGSAP in PBMCs on idiopathic pulmonary arterial hypertension has been examined by investigators [31]. The expression profiles of circRNAs in PBMCs from retinopathy of prematurity patients were revealed by Li et al. through microarray analysis [32]. Additionally, evidence from numerous research points to a potential link between the expression of circRNAs and the incidence and progression of rheumatoid arthritis [33, 34]. Extensive research has shown that circRNA can be considered as a potential biomarker for clinical diagnosis and treatment of CAD [35].

In this research, we found that 1352 circRNAs were significantly differentially expressed (|log2FC |≥ 2 and *p < 0.05*) in patients with CAD, compared with controls. As revealed by the RNA-seq results, the number of up-regulated circRNAs was similar to circRNAs exhibiting downregulation. This finding differs from Yu et al.’s study by which concluded that more down-regulated circRNAs were identified in patients with CAD [36]. This inconsistency could be attributed to the difference in sequencing instruments. We used an illumina Novaseq 6000 instrument with 150 bp paired-end reads to complete our library sequencing, while they were sequenced on an illumina HiSeq 2500 platform with 125-bp paired-end reads. In addition, a large proportion of the circRNAs were represented as exonic in our study, similar to that found by Li et al. [37]. Besides, we discovered that circRNAs were widely distributed throughout all human chromosomes, with chr1, chr2, and chr4 at the top3 of circRNA-containing chromosomes. Surprisingly, we found that there are 29 circRNAs positioned on chrM. It is possible, therefore, that these circRNAs regulate the entry of proteins into the mitochondria, thus participating in metabolic inflammatory processes such as the production of inflammatory factors [38, 39]. What’s more, there are 472 circRNAs were correlated with cardiac hypertrophy, which indicates that they may participate in protein regulation synthesis in cardiomyocytes.

Apart from that, we performed a joint analysis of RNA-seq results in human coronary artery segments and peripheral PBMCs in CAD patients. In total, there are 34 circRNAs that have attracted our attention. The sanger sequence results revealed that no junction sites were found in 30 circRNAs, contrary to some previous studies [40–43]. According to their study, hsa_circFKBP8, hsa_circLMTK2, hsa_circMRRF, and hsa_circCLASP2 were further researched. The difference may be related to the primer design, and redesigning the primers might aid in our understanding of these circRNAs.

Additionally, we constructed the circRNA-miRNA-mRNA networks for hsa_circRPRD1A, hsa_circHERPUD2, hsa_circLMBR1, and hsa_circDHTKD1, aiming to explore the possible regulation mechanism in patients with CAD. It is interesting to note that the PRKX [44] and TFRC [45] expression was downregulated in CAD patients which has already been reported. Moreover, RPS3A, BCL6, and S100B play a critical role in coronary atherosclerosis [44–47]. This finding broadly supports other studies in this area linking circRNAs with CAD, and provides evidence that hsa_circRPRD1A, hsa_circHERPUD2, hsa_circLMBR1, and hsa_circDHTKD1 take part in the pathogenesis of atherosclerosis.

Given that hsa_circRPRD1A, hsa_circHERPUD2, hsa_circLMBR1, and hsa_circDHTKD1 were dysregulated in patients with CAD, we sought to further investigate alterations in hsa_circRPRD1A, hsa_circHERPUD2, hsa_circLMBR1, and hsa_circDHTKD1 in CAD. As expected, the expression levels of hsa_circHERPUD2, hsa_circLMBR1, and hsa_circDHTKD1 were decreased in CAD patients. However, only the differential expression of hsa_circHERPUD2 was statistically significant. qRT-PCR also revealed that the hsa_circRPRD1A expression was significantly decreased in CAD patients compared with controls, which was contrary to expectations. This discrepancy could be attributed to that we had sequenced only ten samples, while we validated in 305 samples. We proceeded with post-hoc analysis for the validation group by G*Power and the power (1-β error prob) was calculated as 0.931, indicating that our experimental results are plausible. Zhang et al. validated 58 genes, 50 of which were consistent in qPCR and RNA-Seq sequencing results, suggesting that our discrepancy is acceptable [48].

To understand the diagnosis ability of hsa_circRPRD1A and hsa_circHERPUD2 for CAD, further statistical analyses were performed. Based on spearman rank correlation analysis and ROC analyses, hsa_circRPRD1A and hsa_circHERPUD2 could be potential diagnostic biomarkers for CAD. Furthermore, univariate and multivariable logistic regression analyses identified hsa_circRPRD1A as a protective factor for coronary artery disease, with the elevated expression of hsa_circRPRD1A1 and a possible decrease in CAD incidence of 37.5%. It’s interesting to note that although the expression of hsa_circDHTKD1 was not statistically different between patients with CAD and normal control subjects, ANOVA found that its expression was different between patients with ordinary and severe CAD. This indicates that although hsa_circDHTKD1 is not a suitable biomarker for diagnosing CAD, its expression still affects the progression of CAD. And it is capable of evaluating the severity of a disease based on the expression of hsa_circDHTKD1.

According to prior studies, light-to-moderate alcohol consumption was particularly protective against CAD [49, 50], which has been attributed to the connection between alcohol consumption with high-density lipoprotein cholesterol [51–53]. According to the additive model, crossover analysis found an antagonistic relationship between the expression of hsa_circHERPUD2 in subjects with CAD and alcohol consumption in our study. The value of RERI was 0.707, indicating that hsa_circHERPUD2 expression interacted with alcohol consumption accounted for 70.7% of the effects induced by hsa_circHERPUD2 expression and alcohol consumption. Although the expression of hsa_circHERPUD2A was not an independent protective factor for CAD, the combined effect of hsa_circHERPUD2 expression and alcohol consumption on the development of CAD is meaningful. An implication of this is the possibility that hsa_circHERPUD2 is more relevant for the development of CAD in individuals with risk factors. Nevertheless, alcohol is not recommended for preventing myocardial ischemia in our study, due to its addictive nature as well as its cancer-causing properties. This suggested that we should also be aware of the influence of classical cardiovascular risk factors in our search for molecular mechanisms of CAD.

Simultaneously, there was no significantly interaction between the hsa_circRPRD1A1 expression and classical cardiovascular risk factors in patients with CAD. However, with a smaller sample size in the control group than in the coronary group, caution must be applied, as such imbalance may lead to overly wide confidence intervals and an unstable combined effect of hsa_circRPRD1A1 expression and classical cardiovascular risk factors. According to these data, we can still infer that the expression of hsa_circRPRD1A1 and hsa_circHERPUD2 are tightly associated with the development of CAD. The interaction effect between circRNAs and classical cardiovascular risk factors in patients with CAD cannot be ignored.

Our study adds to the accumulating evidence that the expression of circRNAs can influence the occurrence and development of CAD, possibly through the regulation of protein entry into mitochondria or ceRNA mechanisms. At the same time, hsa_circRPRD1A and hsa_circHERPUD2 were proven to work as biomarkers for the CAD diagnosis in our study. What’s more, our findings might provide epidemiological support for the independent associations of circRNAs and environment factors and the interactions between circRNAs and environment factors. There are some limitations in our study, nevertheless. Our subjects were a homogenous group of the southern Han Chinese population; hence, it is doubtful whether our results are applied to other groups. Likewise, our study only included 305 individuals, which may be too few to extend this conclusion. Thus, further studies with large populations are needed.

## Conclusion

Coronary artery disease leads to the most morbidity and mortality worldwide nowadays, while most diagnosis examinations are invasive or radiation intensive and difficult to accept by patients. In order to explore potential biomarkers for early CAD diagnosis, we performed high-throughput RNA-seq and qRT-PCR. As the research has demonstrated, hsa_circRPRD1A, hsa_circHERPUD2, hsa_circLMBR1, and hsa_circDHTKD1 caught our attention and were further investigated. The circRNA-miRNA-mRNA network was then constructed based on the rank of the top 5 miRNAs and the 66 mRNAs they are related to. To validate the expression level of hsa_circRPRD1A, hsa_circHERPUD2, hsa_circLMBR1, and hsa_circDHTKD1, a qRT-PCR assay in a large cohort was performed. We can see that the expression of hsa_circRPRD1A and hsa_circHERPUD2 significantly decreased in CAD patients compared with controls. In-depth statistical analyses infer that they could be used as biomarkers for the CAD diagnosis. What’s more, crossover analysis confirmed that the interaction effect between circRNAs and environment factors on patients with CAD cannot be ignored. Our findings might provide epidemiological support for the independent association between circRNAs and classical coronary risk factors as well as their interactions. Future research will concentrate on the significant role that hsa_circRPRD1A and hsa_circHERPUD2 play in CAD pathogenesis.

## Electronic supplementary material

Below is the link to the electronic supplementary material.

Supplementary Material 1

Supplementary Material 2

## Data Availability

CloudSeq Biotech (Shanghai, China) performed high throughput transcriptome sequencing. RNA libraries were constructed by NEBNext® rRNA Depletion Kit (New England Biolabs, Inc., Massachusetts, USA) and TruSeq Stranded Total RNA Library Prep Kit(Illumina, USA). Library quality control and quantification using the BioAnalyzer 2100 instrument (Agilent Technologies, USA). Library sequencing was performed on an Illumina Novaseq 6000 instrument, eventually. The datasets used and analyzed during the current study are deposited in the GEO database with the accession number GSE205255. The resources can be viewed at https://www.ncbi.nlm.nih.gov/geo/query/acc.cgi?acc=GSE205255 with the reviewer secure token: cpwncewgbdmrpcr.

## Abbreviations

CAD: Coronary artery disease
circRNA: circular RNA
ROC: receiver operating characteristic curves
AUC: the area under receiver operating characteristic curves
PBMCs: peripheral blood mononuclear cells
qRT-PCR: quantitative real-time polymerase chain reaction
RPRD1A: regulation of nuclear pre-mRNA domain containing 1 A
HERPUD2: HERPUD family member 2
LMBR1: limb development membrane protein 1
DHTKD1: dehydrogenase E1 and transketolase domain containing 1
