# Supplementary material for: Interaction between the expression of hsa_circRPRD1A and hsa_circHERPUD2 and classical coronary risk factors promotes the development of coronary artery disease
Source: BMC Med Genomics. 2023 Jun 14;16:131. doi: 10.1186/s12920-023-01540-9 (PMC10265751; doi:10.1186/s12920-023-01540-9)
Supplement: Supplementary file 1 — Supplementary Material 1 [file 12920_2023_1540_MOESM1_ESM.docx]

| lncRNA | Forward primer | Reverse primer |
| --- | --- | --- |
| GAPDH | GTCTCCTCTGACTTCAACAGCG | ACCACCCTGTTGCTGTAGCCAA |
| hsa_circRPRD1A | AATGGTAGAGGATGCGTGTATG | TTGGCTAGGTAGAGAAAAGTAAGC |
| hsa_circHERPUD2 | CACAAGCACAAACTGACCAAG | TGGATTTTGGAGAACTGGGAG |
| hsa_circLMBR1 | TTTGAGCACGTTCACTCTCGC | AGTAGGAAACAACGTAGAGAATGG |
| hsa_circDHTKD1 | CCGTTGGATTCTTTACAGCAAG | TTTTGGCAGCTTTATGACCATG |

Supplementary table 1: The oligonucleotide sequences of the primers for qRT-PCR.
