# Supplementary material for: Interaction between the expression of hsa_circRPRD1A and hsa_circHERPUD2 and classical coronary risk factors promotes the development of coronary artery disease
Source: BMC Med Genomics. 2023 Jun 14;16:131. doi: 10.1186/s12920-023-01540-9 (PMC10265751; doi:10.1186/s12920-023-01540-9)
Supplement: Supplementary file 2 — Supplementary Material 2 [file 12920_2023_1540_MOESM2_ESM.docx]

Supplementary table 2. Clinical characteristics of drinkers and non-drinkers in the study population.

| Variables | Non-drinker (n=212) | Drinker (n=79) | *P value* |
| --- | --- | --- | --- |
| Gender(M/F) | 128/84 | 76/3 | **<0.001**** |
| BMI (kg/m2) | 25.027±3.708 | 25.133±3.031 | 0.659 |
| SBP (mmHg) | 130.510±17.784 | 130.680±16.895 | 0.596 |
| DBP (mmHg) | 75.000 (69.250-84.000) | 77.000 (71.000-85.000) | 0.253 |
| Total cholesterol(mmol/L) | 3.620 (3.080 -4.430) | 3.740 (3.040-4.418) | 0.964 |
| TG (mmol/L) | 1.240 (0.930 -1.680) | 1.435 (0.958-1.925) | 0.074 |
| HDL-C (mmol/L) | 1.020 (0.870 -1.170) | 0.940 (0.830-1.080) | **0.026*** |
| LDL-C (mmol/L) | 2.110 (1.750 -2.690) | 2.185 (1.648-2.720) | 0.855 |
| Lp (a) (mg/L) | 169.000 (76.000-395.000) | 145.000(77.000-275.000) | 0.593 |
| Gensini score | 37.500 (13.625 -86.000) | 38.000 (18.000-92.000) | 0.546 |
| hsa_circRPRD1A | 0.470 (0.230 -0.754) | 0.472 (0.204-0.613) | 0.371 |
| hsa_circHERPUD2 | 0.766 (0.450 -1.749) | 0.632 (0.370-1.199) | 0.104 |
| hsa_circLMBR1 | 1.267 (0.488 -3.608) | 1.223 (0.541-2.594) | 0.551 |
| hsa_circDHTKD1 | 1.143 (0.711 -2.902) | 1.268 (0.522-4.492) | 0.650 |

***^*^****P<0.05,* ***^**^****P<0.001.* BMI: body mass index, SBP: systolic blood pressure, DBP: diastolic blood pressure, TG: triglyceride, HDL-C high-density lipoprotein cholesterol, LDL-C: low-density lipoprotein cholesterol, Lp (a): lipoprotein (a). Normally distributed presented by Mean ± SD determined by t-test, the skewness distribution parameters were presented as median (interquartile range) and determined by the Wilcoxon-Mann-Whitney test.
